# The negligible biological impact of reproductive history on aging: a high-resolution variance analysis

**DOI:** 10.3389/fragi.2026.1888676

**Published:** 2026-07-20

**Authors:** Marco Roccetti

**Affiliations:** Department of Computer Science and Engineering Bologna, University of Bologna, Bologna, Italy

**Keywords:** biological determinism, DNA methylation, effect size, epigenetic clocks, reproductive history, stochastic noise, variance analysis

## Abstract

Modern epigenetics increasingly employs biological clocks to quantify the impact of women’s reproductive history on aging, with a very recent study claiming that childbearing significantly decelerates biological senescence in women. This article critically re-examines these claims, challenging the growing trend of biological determinism imposed on reproductive choices. By performing a secondary upper-bound analysis on demographic and epigenetic data, we quantify the predictive power of reproductive history relative to the dominant influence of genetic and stochastic factors. Our analysis reveals that a reported 1-year acceleration in biological aging for women without children, while statistically significant in massive cohorts, possesses negligible explanatory power. When evaluated against the standard deviation of high-longevity populations, this 1-year shift corresponds to ∼1.2% of variance scale in female lifespan, leaving 98.8% governed by stochastic noise. With a standardized effect size near zero (*d* = 0.11) and a survival distribution overlap of 95.6% between groups, the epigenetic signals attributed to childbearing are shown to be practically irrelevant for individual life expectancy. These findings demonstrate how microscopic p-values in large-scale datasets can alter scientific reality thus generating unjustified societal anxiety. This work calls for a return to scientific realism in biometrics, distinguishing between molecular *non-sequiturs* and tangible health outcomes to prevent the pathologization of reproductive trajectories and defend the fundamental stochastic freedom of human life (i.e., the inherent randomness and unpredictability of human lifespan).

## Introduction

Modern epigenetics increasingly relies on biological clocks to quantify the impact of life choices on human aging. While these advancements hold promise, a problematic trend has emerged: the tendency to leverage large-scale genomic datasets to assign biological penalties (or rewards) to individual reproductive choices. This article critically re-examines the statistical foundation of this growing determinism, arguing that current interpretations often conflate mathematical significance with clinical or biological reality (Grissom and Kim, 2012).

The investigation into how reproductive life history shapes the human aging process is deeply rooted in the disposable soma theory of aging, first proposed by Thomas Kirkwood in 1977 (Kirkwood, 1977). This evolutionary framework posits a fundamental conflict in resource allocation: organisms possess a finite pool of metabolic energy that must be strategically partitioned between reproductive effort and somatic maintenance (DNA repair, antioxidant defense, and protein folding). From this perspective, the soma is essentially disposable; its only biological purpose is to ensure the survival of the individual until successful reproduction is achieved. Consequently, every reproductive event, from the physiological demands of gestation to the metabolic toll of lactation, should theoretically incur a cumulative biological debt. According to this model, resources diverted to the germline are resources stolen from the longevity-promoting mechanisms that forestall senescence.

For decades, this trade-off was studied primarily through demographic proxies, such as parity (the number of children borne) and the age at first or last birth. However, the empirical results in human populations remained of limited impact or controversial in nature. In fact, while some large-scale epidemiological studies identified a survival penalty for high parity (the maternal depletion hypothesis), others, particularly in high-longevity Western cohorts, suggested a protective effect of motherhood, where the hormonal or social benefits of childbearing seemed to correlate with increased lifespan. This demographic impasse was attributed to the overwhelming influence of confounding variables, such as socio-economic status, healthcare access, and the healthy mother effect, which historically made it impossible to isolate the pure biological cost of reproduction from the noise of lived experience.

Recently, the emergence of DNA methylation (DNAm) clocks has fundamentally revolutionized this field, promising to move beyond the limitations of chronological age and demographic proxies (Horwath and Raj, 2018). These biomarkers quantify biological aging by measuring the methylation levels of specific cytosine-phosphate-guanine (CpG) sites across the genome. Unlike chronological age, which is a simple measure of elapsed time, these epigenetic clocks offer a high-resolution molecular readout of cellular senescence, supposedly capturing the cumulative impact of environmental stressors and life events. The allure of these clocks lies in their perceived ability to bypass the stochastic noise of individual life expectancy, providing a standardized metric of biological decay that can be compared across diverse populations.

In this specific field of reproductive history, the application of high-throughput epigenetic analyses to massive longitudinal cohorts has sought to identify a definitive molecular signature of parity. Findings in recent literature suggest that reproductive choices are associated with a quantifiable shift of the epigenetic clock. Notably, some studies have reported that each additional child may shift a woman’s biological age forward by approximately 0.5–1.2 years (Ryan et al., 2018; Pollack et al., 2018). Conversely, recent high-impact research, such as the study by Hukkanen et al. (Hukkanen et al., 2026) on the Finnish Twin Cohort, has identified a U-shaped relationship where both high parity and nulliparity are associated with accelerated biological aging. Specifically, Hukkanen et al. suggest that women not having children (nulliparous) undergo an accelerated biological aging of approximately 1.0 years compared to those with a moderate reproductive history (Hukkanen et al., 2026).

While these statistically significant results are presented as molecular evidence for the disposable soma theory, they have also catalyzed a new trend of biological determinism. These molecular shifts are framed as direct predictors of aging, often without accounting for the massive background variance that defines the actual human lifespan.

All this premised, as researchers in the field of medical data analysis, we maintain that scientific integrity requires a rigorous distinction between a statistically detectable signal in a large cohort and a meaningful outcome for the individual. We contend that the current focus on microscopic p-values in massive datasets may distort the scientific landscape. Whether the epigenetic clock suggests an acceleration or a deceleration of 1 year (or slightly more) and regardless of whether this shift is attributed to having many children or having none, the core issue remains the same: the magnitude of the effect. By re-analyzing the normalized signal proportion (upper-bound estimate) by reproductive history in current high-impact literature, our study demonstrates that these claims often lack the effect size necessary to support the deterministic narratives currently being constructed.

Our objective is thus not to challenge the molecular data itself, but to evaluate the quantitative interpretability of the reported epigenetic signals within the broader stochastic variability of human lifespan. While prior work has cautioned against over-reliance on p-values, this study provides a quantitative framework, using variance partitioning, Cohen’s d, and overlap coefficients, specifically for reproductive history claims, and demonstrates the application of an upper-bound signal proportion analysis to epigenetic data. Even under the strongest possible interpretation of the epigenetic signal as a direct survival displacement, the resulting effect remains in fact extremely small relative to the natural population variance, raising questions about its practical and biological interpretability. In fact, a fundamental contradiction plagues modern high-impact publishing. While these studies aim to analyze the specific contribution of life events to lifespan, our analysis will provide evidence that this contribution, focusing specifically on the 1.0-year acceleration reported for nulliparous women (Hukkanen et al., 2026), is statistically indistinguishable from zero in any practical sense. We posit that to speak of a meaningful contribution when the individual risk may be effectively as contained as a 53.1% coin toss (as we are going to demonstrate) is mathematically excessive.

To be further clear: a 1.2% normalized signal proportion (upper-bound estimate) attributed to reproductive history means that for every 100 days of difference in lifespan, 99 days are determined by everything else—genetics, environment, choices, or simple luck, and only 1 day is linked to the factor in question. To build a public health narrative on such a microscopic sliver of data is not just an exaggeration; it is a mathematical insignificance that ignores the overwhelming 98.8% prevalence of human reality. For example, would any of us invest our life’s savings based on information that covers only 1% of the total risk? Why, then, do we allow such a significant shift in risk perception regarding women’s choices based on the same minimal probability?

Beyond this provocative question, our study addresses the issue by focusing specifically on the 1.0-year shift reported as a biological penalty for nulliparous women (Hukkanen et al., 2026). By using the entire Italian female population (2024) as a robust demographic proxy, we will employ variance partitioning techniques and effect size measurements to determine to what extent such a displacement is biologically determinant or, conversely, statistically negligible within the broader context of human longevity.

## Materials and methods

In this Section, we provide all the necessary details on the data and methods used in this study, allowing readers to easily replicate our findings.

### Sources of data

Before exposing the data, we wish to acknowledge a fundamental mechanic of modern biostatistics: with a large enough sample size (*n*), even the smallest biological signal can reach high statistical significance (p < 0.0001), large-sample analyses may in fact amplify statistically detectable but clinically negligible effects. For instance, to promote a clinically minor shift of 6 months ( Δ = 0.5) to a highly significant discovery in a population with a standard deviation of 9.1 years ( σ ), a sample of approximately 10,000 subjects is sufficient, as shown in the Equation 1 developed below:

$$n={\left(3.89\times 9.10.5\right)}^{2}≅5,012\;subjects\;per\;group$$ (1)

The motivation for the 3.89 value is as follows. To evaluate the statistical sensitivity inherent in modern massive cohorts, we derive an empirical factor *z* (tot), calculated as *z* (tot) = $\sqrt{n}\;\Delta /\sigma$ . Conventional power analysis for a two-tailed test ( α = 0.05, β = 0.20) would use a standard threshold of *z* (tot) = $z_{\alpha /2}$ + $z_{\beta }$ = 1.96 + 0.84 = 2.80, which corresponds to a required sample size of approx. 2,600 individuals per group. However, for studies reporting an effect of Δ = 0.5 years with a standard deviation of σ = 9.1, a cohort size of n approx. 5,012 individuals per group [selected to emulate the sample size of (Grissom and Kim, 2012)] yields a sensitivity factor of 3.89. This value highlights that these datasets operate well beyond standard statistical power requirements, allowing for the detection of clinically negligible shifts simply by virtue of their massive sample size. Moreover, to achieve the result above we have reasonably assumed that mortality within a specific age-window (50–70 years) can be locally approximated as a homoscedastic Normal distribution, where any biological shift Δ acts as a linear displacement of the mean without deforming the population variance σ^2^ (Grissom and Kim, 2012). For small perturbations relative to population variance (Δ ≪ σ), such linear Gaussian approximation remains appropriate for effect-size interpretation, even when the full long-term mortality dynamics may follow more complex non-linear models (Abrams and Ludwig, 1995). But the real problem here is that, while these results are statistically valid and potentially interesting for future longitudinal research, they often lack immediate clinical weight for the individual patient. While we confirm to remain open to the possibility that epigenetic shifts are precursors to future health trends, we posit that these signals should be linked to a broader demographic context.

To obtain the results we will expose in the next Section, we have used data from the Italian Institute for Statistics (ISTAT, 2024 mortality tables) as a robust proxy for high-longevity European female populations. In particular, using that data, we have observed a standard deviation ( σ ) in survival of 9.1 years for women at age of 50. It should be noticed that demographic and mortality data used in this study are publicly available from ISTAT 2024 mortality tables, at the URL referenced as (ISTAT Istituto Nazionale di Statistica, 2024). The specific datasets used are the “Tavole di mortalità della popolazione residente (2024)”. All mathematical derivations and statistical calculations performed on these public data are fully described in the remainder of the manuscript to ensure reproducibility.

### Statistical techniques

This study has developed a quantitative secondary analysis to evaluate the biological relevance of epigenetic aging signals reported in (Hukkanen et al., 2026). Specifically, our analysis should be interpreted as an upper-bound quantitative framework where the epigenetic age acceleration reported in previous studies was conservatively treated as a direct displacement of chronological survival, thereby maximizing its potential contribution to lifespan variance. This assumption intentionally favors the strongest possible interpretation of the reported biological signal.

To distinguish between statistical significance and scientific realism, the following mathematical framework was applied as based on the use of the following techniques. First, we used variance partitioning, specifically utilizing the principle of analysis of variance, as pioneered by R.A. Fisher (RA Fisher et al., 1992), to decompose the total observed variability in female lifespan. By comparing the squared biological shift ( $\Delta^{2}$ ), attributed to reproductive history, against the total population variance ( $\sigma^{2}$ ), we calculated a normalized signal proportion (*S*). This metric provides a normalized estimate of the signal magnitude relative to total lifespan variability under the assumed Gaussian displacement framework, effectively separating the signal (reproductive history) from the overwhelming stochastic noise of human vital dynamics.

Then, to assess the magnitude of the epigenetic effect independently of the sample size we adopted the standardized effect size calculating the Cohen’s *d* coefficient (Cohen, 1988). Unlike p-values, which only indicate the likelihood that an effect exists, the effect size measures its practical weight. A Cohen’s *d* is computed as the ratio of the difference between means ( Δ ) and the standard deviation ( σ ). This allows one to determine if the reported biological aging acceleration is a large effect or a negligible one that remains submerged within natural population variability.

Finally, to translate abstract statistical figures into tangible risks, we resorted to the use of Z-score and Cumulative Distribution Function (CDF), first converting the effect size into a Z-score (Sullivan and Feinn, 2012). The Z-score represents the distance between two individuals (e.g., nulliparous vs. others) in terms of standard deviations. Instead, by applying the Cumulative Distribution Function (CDF), denoted as $\Phi \left(z\right)$ , we calculated the actual probability (*P*) that an individual from one given group will experience a shorter lifespan than an individual from another. This transformation turns a statistically significant finding into a practical probability of outcome.

Ultimately, we calculated the so-called Overlapping Coefficient (OVL) to visualize the similarity between population distributions. The OVL measures the area shared by two probability density functions. A high OVL (near 100%) indicates that, despite a statistically detectable difference in means, the two groups are functionally and biologically identical for the vast majority of the population. This metric serves as a final check against the distortions of large-sample datasets, ensuring that scientific conclusions remain linked to observable reality (Inman and Bradley, 1989).

## Results

This section quantifies the impact of the reported 1.0-year epigenetic shift (Hukkanen et al., 2026) relative to the natural stochasticity of human lifespan. By using the high-resolution mortality data of the Italian female population as taken from the Italian Institute for Statistics (ISTAT, 2024 mortality tables) as a robust demographic proxy for high-longevity European female populations (average standard deviation σ = 9.1 years), we evaluate whether such a signal constitutes a deterministic biological penalty or a statistically negligible fluctuation (Abrams and Ludwig, 1995).

### Variance analysis

By applying a variance-analysis framework to evaluate the impact of the reported 1.0-year epigenetic shift ( Δ = 1.0) against the ISTAT population data ( σ=9.1 years ), we estimate an upper bounded normalized contribution of the reported mean shift relative to total demographic variance represented in Equation 2 (ISTAT Istituto Nazionale di Statistica, 2024):

$$S=\frac{\Delta^{2}}{\sigma^{2}}=1/82.81≅0.012=1,2\%.$$ (2)

Under the assumed Gaussian displacement framework, the reported biological shift represents only ∼1.2% of the total demographic variance scale. This also means that the contribution of the reproductive history remains extremely small relative to the intrinsic demographic expectancy for the female population (at least using the Italian data). This implies that for an individual woman, 98.8% of her biological destiny remains governed by factors far more influential than her reproductive history. While this 1% may be a valuable seed for future biological theories, it remains a whisper amidst the 98.8% amplitude of genetics, lifestyle, and stochastic factors that truly define a woman’s lifespan. As already anticipated, it should be reminded that for small perturbations relative to population variance, linear Gaussian approximation remains appropriate for effect-size interpretation and statistically *S* should not be interpreted as a regression-derived coefficient of determination but as an upper bound normalized signal proportion on the variance scale.

### Standardized effect size

To measure how the signal ( Δ ) weighs against the noise of natural human variation ( σ ) we computed the standardized *effect size* (Cohen’s *d* coefficient) (RA Fisher et al., 1992). This coefficient tells us how much the signal ( Δ=1.0 ) weighs against the noise of natural human variation as per Equation 3 ( σ=9.1 ):

$$d=\frac{\Delta }{\sigma }=1/9.1≅0.112$$ (3)

In biostatistics, a Cohen’s *d* coefficient of 0.11 is considered negligible. It means that the effect is so small that it is completely submerged by the 9.1-year standard deviation found in the aforementioned demographic data.

### The probability of outcome

To translate these metrics into a practical answer for the individual, we converted the effect size into a probability of survival displacement. Accepting the linear assumptions of (Hukkanen et al., 2026) and a standard Normal Distribution, we transform the effect size into a *Z*-score (the standard score that measures the distance between two individuals) and then into a probability using the Cumulative Distribution Function ( Φ ) by means of the two following Equations 4, 5 (Cohen, 1988):

$$z=\frac{\Delta }{\sigma \;\sqrt{2}}=d\;/\;\sqrt{2}≅0.11\;/\;1.41≅0.077$$ (4)

$$P=\Phi \left(z\right)=\Phi \left(0.077\right)≅53.1\%$$ (5)

This result is revealing: even under the best-case assumption that a 1-year epigenetic acceleration translates directly to a 1-year reduction in chronological lifespan, the probability that a nulliparous woman will experience a shorter lifespan compared to the comparison group is only 53.1% (under a gaussian equal-variance assumption). More technically, even accepting the strongest interpretation of the epigenetic signal, the probability that a randomly selected woman from one group exhibits a lower survival outcome than a randomly selected woman from the comparison group is very low.

Therefore, while the 1.0-year signal produced is statistically significant, its clinical impact (*d*) is near zero. We feel also the duty to say that a 1-year acceleration in epigenetic aging does not necessarily translate into a 1:1 proportional reduction in actual chronological lifespan. Nonetheless, our analysis remains robust, precisely because it has evaluated that epigenetic signal against the maximum possible resolution of human mortality variance. By demonstrating that even a literal 1-year shift in life expectancy would be submerged by the stochastic noise of high-longevity populations, we can maintain that any subtler or non-linear biological effect would, *a fortiori*, be even more indistinguishable from the background variance of human vital dynamics.

### Functional identity: distribution overlapping

The final confirmation of this negligible impact comes from the Coefficient of Overlapping (OVL) between two groups (nulliparous vs. others) whose computation of Equation 6 indicates that the survival trajectories of women with and without children are 95.6% identical:

$$OVL=2\Phi \;\left(-\frac{\left|d\right|}{2}\right)≅2\Phi \left(-0.055\right)≅95.6\%$$ (6)

where Φ denotes the standard normal cumulative distribution function (CDF). Here, the high degree of similarity is numerically evident, showing that the two groups are functionally identical for the vast majority of the population. Table 1 summarizes these metrics, contrasting the statistical significance found in large-scale studies with their negligible clinical and practical impact.

**TABLE 1:** Summary of results.

Statistical metric	Numerical value	Biostatistical interpretation
Reported biological shift Δ	1.0 Year	Statistically significant signal in massive cohorts (p < 0.001)
Population standard deviation σ	9.1 Years	Natural stochastic variability of human lifespan (ISTAT 2024 mortality tables)
Normalized signal proportion: *S*	1.2%	Practical impact assessment^1^
Standardized effect size (Cohen’s *d*)	0.11	Negligible effect; signal submerged by noise
Probability of outcome: *P*	53.1%	Practical risk; equivalent to a coin toss
Overlapping coefficient: *OVL*	95.6%	Functional identity between groups

1 This is a normalized signal proportion (upper-bound estimate) and not an *R*2; the actual variance explained would be 1.1% using *R* ^2^ = *d* ^2^/(4 + *d* ^2^).

## Discussion

We maintain that the issue here is not a debate over whether the epigenetic links are causal. Quite frankly, even if causal, any effect may remain quantitatively negligible if the magnitude of that effect is invisible to the naked eye of reality. Whether the link is causal or merely associative, our analysis demonstrates that its normalized magnitude corresponds to only ∼1.2% of the total survival variance scale, leaving a 95.6% overlap between the two groups of women with different reproductive histories. To use such a microscopic sliver of data to construct a narrative about women’s aging is not just a statistical error; it is an over-interpretation of the available data.

The pervasive emphasis on statistical significance over effect magnitude risks to create a science that is technically significant but practically elusive, in the sense that large-sample analyses may amplify statistically detectable but clinically negligible effects. By choosing to prioritize p-values over the actual magnitude of the effect, modern science suggests that a molecular minimality is more important than the tangible complexity of a woman’s life. When we interrogate large datasets (n > 10,000) until we yield a p-value of 0.0001 for a 1% effect, we are not only discovering truth; but also inflating statistics.

As scientists who have seen the evolution of these methods, we have again to state the obvious: we cannot allow biostatistics to become a tool for a new form of biological determinism. Science should not be used to label reproductive choices with unjustified warnings of accelerated/decelerated decay when, even under the best-case assumption that a 1-year epigenetic acceleration translates directly to a 1-year reduction in chronological lifespan, the individual risk remains a 53.1% coin toss. A 1.0-year shift in life expectancy is a signal that is still submerged by the stochastic noise of high-longevity populations; it is indistinguishable from the background variance of human vital dynamics, a critical issue further explored in (Roccetti, 2026), where we demonstrate how the inflation of statistical significance often comes at the expense of biological plausibility in clinical reality.

We posit that it is time for the scientific community to distinguish between a molecular non sequitur and a tangible, life-changing reality. Science should not be a mechanism for determining biological penalties/rewards on the freedom of human choice; it should be a tool for measuring the world as it truly is: vast, varied, complex (and largely beyond the reach of a 1.2% estimated signal magnitude).

Importantly, our analysis should not be interpreted as a dismissal of epigenetic research or its potential utility for population-level health tracking. We acknowledge that, even if currently modest, molecular signals may provide valuable insights for public health monitoring and general wellbeing initiatives. Here, we only contend that the premature translation of these signals into deterministic individual narratives remains scientifically premature. While these findings are statistically significant within large-scale demographic datasets, our analysis demonstrates that they remain functionally negligible at the level of individual health risk assessment. This is underscored by the fact that the effect size attributed to reproductive history is less than half that of other recognized risk factors, such as alcohol consumption, for example,; if the latter is already complex to translate into an individual clinical prognosis, the impact of the former appears even more marginal. By integrating these considerations into the broader discourse, we advocate for a methodological recalibration: one that acknowledges the stochastic nature of human life and insists on a clear distinction between population-wide statistical associations and individual health outcomes.

It is also important to notice that for our study, we treat the reported 1.0-year shift as if it were entirely causal (providing an upper-bound estimate). However, in reality, residual confounding by socioeconomic status, health behaviours, or healthcare access could attenuate this signal further. Thus, even the 1.2% signal proportion likely overestimates the true contribution of reproductive history.

Limitations to our study need also to be recognized.

First, our analysis has locally approximated survival within a specific age window (50–70 years) as a homoscedastic Normal distribution. While human mortality across the entire lifespan follows more complex patterns (e.g., Gompertz-Makeham dynamics), a linear displacement of the mean remains a mathematically robust proxy for evaluating effect sizes in high-longevity cohorts. However, we acknowledge that more sophisticated non-linear models could further refine the interaction between epigenetic aging and actual chronological survival.

Second**,** we used demographic data from the Italian National Institute of Statistics (ISTAT 2024 mortality tables) as a proxy for high-longevity European female populations. While Italian women share similar life expectancy and socio-economic profiles with other Western European cohorts, slight variations in environmental or genetic backgrounds across different nations could marginally shift the total variance ( $\sigma^{2}$ ). Nevertheless, the magnitude of the stochastic noise is so dominant in all modern developed societies that the core conclusion, the negligible impact of reproductive history, remains globally applicable.

Third, this analysis specifically focuses on the contrast between nulliparous women and those with children to address the primary narrative of biological penalties associated with childlessness. Consequently, we did not investigate the potential non-linear, U-shaped relationship between parity and longevity often suggested in epidemiological literature, where very high parity may introduce different physiological stressors. However, since the reported epigenetic shift for nulliparous women (1.0 years) is frequently cited as a major finding, our focus on this specific group remains a necessary and sufficient test for the general relevance of such biological signals.

Third, this analysis specifically focuses on the contrast between nulliparous women and those with children to address the primary narrative of biological penalties associated with childlessness. The 95.6% overlap we identified specifically compares nulliparous women against the combined group of all parous women. Because the original study reported a U-shaped relationship, the overlap with a moderate-parity subgroup might differ. Our analysis is a conservative test against the broadest comparison group. Essentially, we did not investigate the potential non-linear, U-shaped relationship between parity and longevity often suggested in epidemiological literature, where very high parity may introduce different physiological stressors. However, since the reported epigenetic shift for nulliparous women (1.0 years) is frequently cited as a major finding, our focus on this specific group remains a necessary and sufficient test for the general relevance of such biological signals.

Conclusively, this study operates on the reported acceleration of epigenetic clocks. It must be noted that a 1.0-year shift in a molecular biomarker does not always translate into a 1:1 reduction in actual lifespan. By treating the epigenetic signal as a direct proxy for survival, our analysis actually grants the original determinist claims the best-case scenario. If the link between these molecular signals and tangible mortality is non-linear or attenuated, the actual explained variance would be even lower than the 1.2% reported here, further confirming the evidence we have brought to the foreground.

This article has challenged the growing trend of using epigenetic clocks to suggest biological determinism on women’s reproductive choices. Whether current epigenetic research claims that childbearing accelerates biological aging or, conversely, that childlessness decelerates it, our study demonstrates that, even under the best-case assumption that a 1-year epigenetic acceleration translates directly to a 1-year reduction in chronological lifespan, such findings, while statistically significant, may remain practically and biologically irrelevant. By applying variance partitioning and Cohen’s effect size to demographic data, we show that reproductive history explains a mere 1.2% of the variance in female lifespan, leaving 98.8% of a woman’s biological destiny governed by stochastic noise and genetics.

We close calling for a balanced dialogue: one that celebrates molecular progress without sacrificing the autonomy of the individual in the face of statistical insignificance. Let us stop over-reliance on p-values to bolster preliminary findings and start respecting the vast, stochastic freedom that defines the human experience. Science is at its best when it acknowledges its limits, rather than pretending that every fluctuation is a mandate for how a human being should live.

## Data Availability

The original contributions presented in the study are included in the article/supplementary material, further inquiries can be directed to the corresponding author.
